# Modulating effects of heat-killed and live *Limosilactobacillus reuteri* PSC102 on the immune response and gut microbiota of cyclophosphamide-treated rats

**DOI:** 10.1080/01652176.2024.2344765

**Published:** 2024-04-29

**Authors:** Md. Sekendar Ali, Eon-Bee Lee, Yixian Quah, Syed Al Jawad Sayem, Muhammad Aleem Abbas, Kyoungho Suk, Seung-Jin Lee, Seung-Chun Park

**Affiliations:** a Laboratory of Veterinary Pharmacokinetics and Pharmacodynamics, Institute for Veterinary Biomedical Science, College of Veterinary Medicine, Kyungpook National University, Daegu, South Korea; b Department of Biomedical Science and Department of Pharmacology, School of Medicine, Brain Science and Engineering Institute, Kyungpook National University, Daegu, South Korea; c Department of Pharmacy, International Islamic University Chittagong, Kumira, Chittagong, Bangladesh; d Developmental and Reproductive Toxicology Research Group, Korea Institute of Toxicology, Daejeon, South Korea; e Cardiovascular Research Institute, Kyungpook National University, Daegu, South Korea

**Keywords:** *Limosilactobacillus reuteri* PSC102, neutrophil migration and phagocytosis, splenocyte proliferation, T lymphocyte differentiation, cytokines, gut microbiota

## Abstract

In the present study, we investigated the potential immunomodulatory effects of heat-killed (hLR) and live *Limosilactobacillus reuteri* PSC102 (LR; formerly *Lactobacillus reuteri* PSC102) in RAW264.7 macrophage cells and Sprague–Dawley rats. RAW264.7 murine macrophage cells were stimulated with hLR and LR for 24 h. Cyclophosphamide (CTX)-induced immunosuppressed Sprague–Dawley rats were orally administered with three doses of hLR (L-Low, M-Medium, and H-High) and LR for 3 weeks. The phagocytic capacity, production of nitric oxide (NO), and expression of cytokines in RAW264.7 cells were measured, and the different parameters of immunity in rats were determined. hLR and LR treatments promoted phagocytic activity and induced the production of NO and the expression of iNOS, TNF-α, IL-1β, IL-6, and Cox-2 in macrophage cells. In the *in vivo* experiment, hLR and LR treatments significantly increased the immune organ indices, alleviated the spleen injury, and ameliorated the number of white blood cells, granulocytes, lymphocytes, and mid-range absolute counts in immunosuppressive rats. hLR and LR increased neutrophil migration and phagocytosis, splenocyte proliferation, and T lymphocyte subsets (CD4^+^, CD8^+^, CD45RA^+^, and CD28^+^). The levels of immune factors (IL-2, IL-4, IL-6, IL-10, IL-12A, TNF-α, and IFN-γ) in the hLR and LR groups were upregulated compared with those in the CTX-treatment group. hLR and LR treatments could also modulate the gut microbiota composition, thereby increasing the relative abundance of Bacteroidetes and Firmicutes but decreasing the level of Proteobacteria. hLR and LR protected against CTX-induced adverse reactions by modulating the immune response and gut microbiota composition. Therefore, they could be used as potential immunomodulatory agents.

## 1. Introduction

The immune system, comprising the spleen, bone marrow, and thymus, is a rigorous defense mechanism against microbes and foreign antigens (Yatim and Lakkis 2015). This highly sophisticated system involves various immune cells, macrophages, and lymphocytes. Lymphocytes secrete cytokines and antibodies that are associated with cell-mediated and humoral immunity. Any disorder in the immune system can result in inflammatory diseases, autoimmune diseases, obesity, diabetes, and even cancer (Rohm et al. 2022). The immune functions of the body are essential for the inhibition of and recovery from these immune-mediated disorders. Therefore, developing a new, effective, and safer immunomodulating agent may be an efficient and effective strategy for the treatment of immunosuppressive disorders.

The microbiota is a diverse and intricate ecosystem comprising trillions of bacteria, including thousands of species in mammals; as such, it is gradually becoming recognized as an integral part of the host immune regulation (Colella et al. 2023). Intestinal bacteria play an important role in maintaining and developing host immunity by triggering the immune system and supporting the barrier functions of the epithelial layer (Håkansson et al. 2015). However, when microbial dysbiosis occurs, it triggers the change in the intestinal epithelial mucosa and stimulates inflammation by external stimuli (Weiss and Hennet 2017).

Researchers have been developing effective ways of using probiotics to treat or prevent immunosuppressive diseases and gut microbiota dysbiosis for years. Several bioactive components of probiotics, such as metabolites, organic acids, short-chain fatty acids, peptides, enzymes, proteins, and exopolysaccharides (EPSs), contribute to comprehensive health benefits, including maintenance of intestinal microbial balance and immunomodulatory effects (Indira et al. 2019; Bernardeau and Cretenet 2019). Moreover, certain active cellular constituents of probiotics, such as peptidoglycans, teichoic acid, lipoteichoic acid, and lipopolysaccharides, have a role in regulating the immune system by promoting both innate and adaptive immune responses (Yeşilyurt et al. 2021). Nevertheless, there is ongoing debate regarding the safety of using live probiotics. Systemic infections are brought on by translocation in pediatrics and susceptible individuals (Kothari et al. 2019). Furthermore, the administration of probiotics may increase the likelihood of gaining antibiotic-resistant genes and disrupt the gut microbiota composition in newborns (Imperial and Ibana 2016). The consumption of live probiotics in specific risk factors, such as diabetes, mitral regurgitation, and short-gut syndrome, has the potential to result in various sepsis, endocarditis, and bacteremia (Boumis et al. 2018; Castro-González et al. 2019; Katkowska et al. 2021). In order to overcome the potential hazards, probiotics are typically used as non-antibiotic-resistant or non-infectious agents, mainly in heat-killed forms. Investigations have suggested that heat-killed probiotics can modulate the immune response. Heat-inactivated *Lactobacillus rhamnosus* ATCC7469 induces the synthesis of tumor necrosis factor (TNF)-α, interleukin (IL)-6, IL-1β, IL-4, IL-10, and IL-12 in RAW264.7 cells (Jorjão et al. 2015). Lee et al. (2019) showed that heat-killed *L. plantarum* LM1004 can elicit immunomodulatory effects by modulating the expression of serum TNF-α, inducible nitric oxide synthase (iNOS), and toll-like receptor (TLR)-4. It was investigated that heat-inactivated *L. gasseri* can boost immune activity by enhancing CD8^+^ lymphocyte levels (Miyazawa et al. 2015). Moreover, heat-killed *L. plantarum* L-137 stimulates immune functions by modulating intestinal microbiota composition (Nakai et al. 2021).

*L. reuteri* is an important probiotic that has been shown to possess various health-beneficial activities, including immunomodulatory effects (Jørgensen et al. 2017; De Marco et al. 2018; M.S. Ali et al. 2022). Additionally, it has the capability to modulate the composition of gut microbiota (Li et al. 2020). Since *L. reuteri* has been described to show beneficial activity in numerous models, we postulate that *L. reuteri* PSC102 possesses immunomodulatory activities. Therefore, we investigated the immunomodulatory effects of heat-killed *L. reuteri* PSC102 (hLR) and live LR in cyclophosphamide (CTX)-induced immunosuppressed Sprague–Dawley rats. Cyclophosphamide (CTX) is widely used as a myelotoxic alkylating agent in creating an immunosuppressive model to examine immunomodulatory activities (Meng et al. 2018; Noh et al. 2019; Zhang et al. 2020).

## 2. Materials and methods

### 2.1. Preparation of sample

*L. reuteri* PSC102 (preserved at the Laboratory of Veterinary Pharmacokinetics and Pharmacodynamics, College of Veterinary Medicine, Kyungpook National University) was cultured in de Man Rogosa Sharpe (MRS) media at 37 °C for 24 h. Biomasses were obtained by centrifugation (6000 × *g* for 10 min) and washed with phosphate-buffered saline. Whole lysates were dried in a vacuum freeze dryer (Operon Co., Ltd., Gyeonggi, South Korea) and kept at −70 °C for the experiment. The colony forming units (CFU) per gram of the dried sample was measured by incubating the sample on an MRS agar plate. The sample was heat-inactivated for 15 min at 80 °C.

Four different temperatures (37 °C, 60 °C, 80 °C, and 100 °C) and time points (0, 15, 30, 45, and 60 min) were set to examine and determine the heat inactivation of *L. reuteri* PSC102. After 24 h of culturing at 37 °C and subsequent centrifugation (6000 × *g*, 10 min), the cell biomasses were washed thrice with PBS. Approximately 1 × 10^9^ CFU/mL of pellets was suspended in PBS and heated in a water bath at different time points and temperatures. After being heated, the bacteria were quickly chilled to 4 °C. A small portion of the heated bacterial suspension was serially diluted and incubated by spreading on MRS agar plates at 37 °C for 24 h. The viable colonies of each sample were obtained and expressed in CFU per milliliter.

### 2.2. Morphological analysis via scanning electron microscopy (SEM)

The morphological features of hLR and LR were analyzed through SEM as described previously (Ali et al. 2023). hLR and control bacterial cells were fixed in 2.5% glutaraldehyde in PBS (pH 7.0) at 4 °C for 2 h and washed thrice with PBS. The cells were washed and dehydrated using a series of ethanol concentrations (30%, 50%, 70%, 80%, 90%, and 100%). The samples were frozen overnight by storing at −70 °C and freeze-dried for 24 h. Then, the samples were mounted on SEM tubes, successively sputter-coated with gold–palladium, and analyzed under a SEM (model S-4300; Hitachi, Japan) at 5.0 kV and magnification of ×35000.

### 2.3. RAW264.7 cell culture and in vitro study

RAW264.7 cells were collected from Korean Cell Line Bank and cultured in Dulbecco’s modified Eagle medium (DMEM, Welgene, Gyeongsangbuk, South Korea) supplemented with 10% fetal bovine serum (FBS) and 1% penicillin and streptomycin (P/S). The cell culture was maintained in an incubator at 37 °C and 5% humidified conditions.

#### 2.3.1. Cell viability assay

For cell viability evaluation, RAW264.7 cells were cultured in a 24-well plate (SPL Life Science Co., South Korea). Then, 1 × 10^5^ cells/well were seeded in a culture plate and incubated at 37 °C in a 5% CO_2_-supplied incubator for 12 h. After the medium was changed, the cells were treated with hLR and LR and incubated for 24 h. The viability of RAW264.7 cells upon sample treatment was measured using a 3-(4,5-dimethylthiazol-2-yl)-2,5-diphenyl tetrazolium bromide (MTT) assay (Quah et al. 2020).

#### 2.3.2. Phagocytic activity

The phagocytic activity of RAW 264.7 cells was assessed using the neutral red uptake method (Zhang et al. 2019). Initially, cells were seeded in a 96-well plate at a density of 1 × 10^5^ cells/mL and subsequently incubated overnight at 37 °C in a humidified atmosphere containing 5% CO_2_. Following the seeding period, cells were subjected to various treatments for a duration of 24 h. Upon completion of the treatment, the supernatant was discarded, and 200 μL of medium containing 20 µL of neutral red was added to each well, followed by a 2-h incubation period. Subsequently, the cells within the 96-well plate were washed with PBS to eliminate any precipitated neutral red. Cell lysis was achieved by the addition of 200 μL of cell lysate (composed of ethanol and 0.01% acetic acid at a 1:1 ratio), and the mixture was maintained at room temperature on a slow shaking plate for 10 min. The optical density at 540 nm was then measured using a microplate reader (BioTek, Winooski, VT, USA).

#### 2.3.3. Production of NO

The level of NO production was assayed using Griess reagent. The cultured RAW264.7 cells (1 × 10^5^ cells/well) were seeded into a 24-well plate for 12 h and treated with hLR and LR for 24 h. The supernatant (100 μL) was collected and mixed with 100 μL of Griess reagent (0.1% N-(1-naphthyl)ethylenediamine dihydrochloride and 1% sulphanilamide in 5% phosphoric acid) in a 96-well plate. The plate was then shaken in an orbital shaker at room temperature for 1 min. Absorbance was read at 540 nm in a multi-mode microplate reader (BioTek, Winooski, VT, USA). NO production was quantified as nitrite (NO_2_) based on a standard curve established from sodium nitrite (NaNO_2_; (Ali et al. 2022).

#### 2.3.4. Quantitative real-time polymerase chain reaction (qRT-PCR)

RAW264.7 cells were treated with samples for 24 h, and their total RNA was extracted using TRIzol reagent (Life Technologies, CA, USA). Then, 5 µg of RNA from each sample was transcribed into cDNA by using AccuPower® RT premix (Bioneer, Daejeon, South Korea). The relative mRNA expression levels of different cytokines such as iNOS, TNF-α, IL-6, IL-1β, and cyclooxygenase (Cox)-2 were assessed using a cycler CFX96 Touch^TM^ PCR detection system (Bio-rad, Singapore). The following particular primers were utilized in PCR amplification, with β-actin as the internal control: iNOS forward (F): TGTGGCTACCACATTGAAGAA and reverse (R): TCATGATAACGTTTCTGGCTCTT; TNF-α F: CTGTAGCCCACGTCGTAGC and R: GGTTGTCTTTGAGATCCATGC; IL-1β F: TGAGCACCTTCTTTTCCTTCA and R: TTGTCTAATGGGAACGTCACAC; IL-6 F: TAATTCATATCTTCAACCAAGAGG and R: TGGTCCTTAGCCACTCCTTC; Cox-2 F: CACTACATCCTGACCCACTT and R: ATGCTCCTGCTTGAGTATGT; and β-actin F: GTCATCACTATTGGCAACGAG and R: TTGGCATAGAGGTCTTTACGG. For real-time qPCR, the following cycling conditions were used: initial denaturation and enzyme activation at 95 °C for 5 min; 40 cycles of amplification at 95 °C for 10 s; and annealing at 60 °C (iNOS and IL-1β) and 56 °C (TNF-α, IL-6, and Cox-2) for 20 s.

### 2.4. Animals and in vivo study

The experimental protocols used for this animal study were approved by the Committee on Care and Use of Laboratory Animals of Kyungpook National University (KNU-2021-50). Four-week-old specific pathogen-free Sprague–Dawley rats were purchased from Orient Bio Inc. (Gyeonggi-do, Republic of Korea). They were acclimatized in a laboratory environment for 7 days under the following conditions: room temperature, 22 °C–26 °C; relative humidity, 55%–70%; and 12 h/12 h light and dark cycle. They were supplied with *ad libitum* filtered water and a standard pellet diet throughout the experiment. They were randomly distributed into eight groups with eight rats each (Table 1). Except for the normal control and levamisole groups, all the groups were intraperitoneally (i.p.) injected with cyclophosphamide (80 mg/kg/day) dissolved in sterile isotonic saline for the first 3 consecutive days to induce immunosuppression. Then, they were administered a daily oral dose of 1 mL of levamisole, LhLR, MhLR, HhLR, and LR dissolved in distilled water for 3 weeks through gavage feeding (Figure S1). Physical examination and body weight measurement were performed regularly for dosage adjustment. On day 21, the rats were terminated, and blood was collected and centrifuged (1000 × *g*, 10 min) to separate serum for enzyme-linked immunosorbent assay (ELISA); their organs were processed accordingly.

**Table 1.** Description of treatment groups.

Groups	Description
Group I	Normal control group: received only water and normal diet
Group II	Levamisole group: received a known immunostimulant (Levamisole, 40 mg/kg/day)
Group III	CTX group: received a known immunosuppresant (Cyclophopshamide, 80 mg/kg/day)
Group IV	Positive control group: received a known immunostimulant (Levamisole, 40 mg/kg/day)
Group V	Treatment group: received low dose (10^6^ CFU/kg/day) hLR (LhLR)
Group VI	Treatment group: received medium dose (10^9^ CFU/kg/day) hLR (MhLR)
Group VII	Treatment group: received high dose (10^11^ CFU/kg/day) hLR (HhLR)
Group VIII	Treatment group: received (10^11^ CFU/kg/day) live LR (LR)

#### 2.4.1. Analysis of body weight and immune organ index

The weight of the rats was measured regularly throughout the experiment to adjust the dose. Before they were sacrificed, their body weight was taken, and their immune organs were excised and accurately weighed. The following formula was used to calculate the immune organ index:

$$\text{Index}(\text{mg}/g)=\frac{\text{Weight of spleen or thymus}}{\text{Body weight}}$$

(Fei et al. 2022).

#### 2.4.2. Spleen histological analysis

Spleen tissues were fixed with 10% of buffered formalin solution. Then, the organs were processed and embedded in paraffin. The samples were cut (4–5 μm thick) using a frozen section machine and stained with hematoxylin and eosin (H&E). The histological characteristics of the tissues were assessed under a microscope.

#### 2.4.3. Complete blood count (CBC) analysis

After final drug administration, blood was collected from the sacrificed rats and placed in a heparinized tube. CBC analysis was performed following previously described procedures (Lee et al. 2018). The number of immune blood cells such as WBCs, granulocytes, lymphocytes, and mid-range absolute, which generally comprises basophils, eosinophils, and monocytes, were counted using URIT-300 Vet Plus (URIT Medical Electronic Co., Ltd., Guangxi, China).

#### 2.4.4. Neutrophil isolation and counting

Histopaque® (Sigma-Aldrich, USA) was used to separate neutrophils from the whole blood. Briefly, a 15 mL conical tube was filled with equal parts of whole blood and Histopque-1077; then, the specimen was centrifuged (400 × *g*, 30 min) at room temperature. After the upper layer was carefully aspirated within 0.5 cm of the opaque interface, the interface was collected, washed with 10 mL of PBS, and centrifuged at 250 × *g* for 10 min. The cells were counted using a hemocytometer by mixing with trypan blue after they were reconstituted in DMEM.

#### 2.4.5. Neutrophil migration assay

A neutrophil migration assay was conducted using a Cytoselect^TM^ 24-well migratory test kit (3 μm, fluorometric format) in accordance with the manufacturer’s instructions (Cell Biolabs, INC. CA, USA). Briefly, after neutrophils were isolated as previously described, 100 mL of 5 × 10^6^ cells/mL in serum-free DMEM was added to the insert, and 500 μL of the media containing 10% FBS was added to the lower well of the migratory plate. The cells were then incubated for 24 h, and the media were aspirated from the inside insert. They were further incubated at 37 °C for 30 min after the insert was transferred to a clean well containing 200 μL of a cell detachment solution. The cells were fully removed from the membrane’s underside by gently tilting the insert into the detachment solution that was then mixed with 400 μL of the media containing migrating cells. The mixture and 60 μL of a 4 × lysis buffer/CyQuant® dye solution (Thermo Fischer Scientific, Waltham, Massachusetts, USA) were poured into a 96-well plate and left to stand at room temperature for 20 min. The mixture (200 μL) was then placed in a 96-well plate to read the fluorescence at 480 nm and expressed as relative fluorescence unit (RFU).

#### 2.4.6. Neutrophil phagocytosis assay

CytoselectTM 96-well phagocytosis assay (zymosan substrate-based; Cell Biolabs, Inc., CA, USA) was performed in accordance with the manufacturer’s instructions to determine the phagocytic activity of neutrophils. Briefly, neutrophils (100 μL of 10^6^ cells/mL) was seeded in a 96-well plate, and 10 μL of zymosan was added to each well. The resulting sample was incubated at 37 °C for 2 h. After centrifugation, the culture medium was removed by aspiration and washed with cold PBS. The fixation solution (100 μL) was supplied to each well, and the wells were left to stand at room temperature for 10 min. Then, the fixation solution was eliminated by centrifugation and washed with PBS. The prepared blocking solution (100 μL) was added to each well and incubated at room temperature for 60 min. After the solution was centrifuged and washed, the prepared permeabilizing solution (100 μL) was added, and the mixture was left to stand at room temperature for 5 min. After the mixture was washed, a detection antibody solution (100 μL) was added to each well. After the solution was washed again with PBS, 50 μL of detection buffer was added to each well and incubated at room temperature for 10 min. A substrate solution (100 μL) was added to each well to initiate the reaction by incubating at 37 °C for 20 min. After 50 μL of stop reagent was added, absorbance was measured at 405 nm by using a microplate reader (BioTek Instruments, Inc., Winooski, USA).

#### 2.4.7. Isolation of PBMCs

Whole blood was collected into anticoagulant-coated tubes and mixed well. An equal volume of whole blood (3 mL) and Histopaque-1077 (Sigma-Aldrich, St. Louis, MO, USA) was mixed in a 15 mL falcon tube to create a density gradient. The upper layer above the opaque interface containing the mononuclear cells was carefully removed after centrifugation at 400 × *g* and room temperature for 30 min. The opaque layer was collected and transferred into a conical tube. The cells were washed with 10 mL of PBS and then centrifuged at 250 × *g* for 10 min. After the supernatant was discarded, the cells were resuspended in 1 mL of FACS buffer. Finally, the cells were counted using a hemocytometer.

#### 2.4.8. Preparation of splenocytes and thymocytes

Splenocytes and thymocytes were prepared in accordance with previously described methods (Ahmad et al. 2015). Briefly, after the rats were terminated, the spleen and thymus were removed aseptically and kept in Hank’s balanced salt solution (HBSS; Gibco Life Technologies, New York, NY, USA). The organs were sieved through a 70 μm nylon cell strainer (BD Bioscience, CA, USA) by using a 3 mL syringe plunger. Then, the cells were centrifuged at 1800 rpm for 5 min, suspended in red blood cell (RBC) lysing buffer to lyse RBCs, and centrifuged again (1800 rpm, 5 min) to remove the lysed RBCs. The supernatant was discarded and washed thrice with PBS. The cell pellets were suspended in Roswell Park Memorial Institute (RPMI)-1640 media (Sigma-Aldrich, St. Louis, MO, USA), and the number of cells was counted using a hemocytometer *via* a trypan blue dye exclusion method.

#### 2.4.9. Splenic lymphocyte proliferation assay

A splenocyte proliferation assay was performed using mitogen concanavalin A (Con A) and lipopolysaccharide (LPS). In this procedure, 100 μL of 1 × 10^6^ cells/mL from different groups was seeded in a 96-well plate containing RPMI-1640 media supplemented with FBS (10%) and P/S (1%). The total volume was adjusted to 200 µL by adding mitogen Con A or LPS (5 μg/mL), and the cells were incubated at 37 °C for 72 h. The MTT reagents (20 μL) of 5 mg/mL were mixed in each well and incubated for 4 h. Then, 200 μL of dimethyl sulfoxide was added to each well, and absorbance was read at 570 nm.

#### 2.4.10. Fluorescence-activated cell sorting (FACS) analysis of T lymphocyte subpopulations

The T lymphocyte phenotyping of the thymus, PBMCs, and spleen was performed at a concentration of 1 × 10^6^ cells/mL by harvesting them into a FACS tube. T cells were stained with the following anti-rat antibodies: anti-CD3^+^ (APC-conjugated), anti-CD4^+^ (PE-Cy7-conjugated), anti-CD8^+^ (FITC-conjugated), anti-CD45RA^+^ (PE-conjugated) and anti-CD28^+^ (BV711-conjugated). Then, 10 μL of 10^6^ cells/mL was mixed and incubated in a dark environment at 4 °C for 30 min. The cells were centrifuged (300× g, 5 min) with FACS buffer to remove unbound antibodies. For FACS analysis, the cells were suspended in 400 μL of FACS buffer. PBMCs, thymocytes, and splenocytes were analyzed in terms of CD3 T cells, CD4 Th, CD8 CTL, CD45RA, and CD28 regulatory T cells (Treg). Multicolor FACS with FACSDiva version 6.1.3 on BD FACSAria^TM^ III (BD Biosciences, San Diego, CA, USA) was used to acquire and analyze the data.

#### 2.4.11. Determination of cytokines in the serum

Cytokine concentrations were measured *via* ELISA in accordance with the manufacturer’s protocol (ELK Biotechnology, Wuhan, China). Briefly, 100 μL of the sample or standard was transferred to each well and incubated at 37 °C for 2 h. The wells were washed thrice with the wash buffer. Then, 100 μL of pre-diluted biotinylated antibody was mixed into each well and incubated at 37 °C for 1 h. The wells were washed three more times with the wash buffer before 100 μL of pre-diluted streptavidin-HRP was added to each well and incubated at 37 °C for 1 h. After three rounds of washing, 90 μL of TMB substrate was added to each well and incubated at 37 °C for 20 min. Lastly, 50 μL of the stop reagent was added to each well, and absorbance was read at 450 nm. Protein concentrations were determined by generating a standard curve, and results were expressed in picograms per milliliter.

#### 2.4.12. Gut microbiota analysis

Five groups (normal control, CTX, positive control, HhLR, and LR) were prepared to analyze the microbiota composition. The HhLR group was selected to represent the hLR treatment groups. Fecal microbial genomic DNA was extracted using a QIAamp DNA stool kit (Qiagen Inc., Hilden, Germany) following the manufacturer’s protocols. The rats’ fecal samples were microbially profiled on the basis of the 16S rRNA gene sequencing amplified by Pacific Biosciences performed in Chun Lab Inc. (Seoul, South Korea). The EzBioCloud Microbiome Taxonomic Profile (MTP) and EZBioCloud 16S database PKSSU4.0 were used to create the MTP from next-generation sequencing (Yoon et al. 2017). UCLUST was used to perform high-quality sequence clustering based on operational taxonomic units (OTUs) with a 97% similarity cutoff. The alpha-diversity (Chao 1, ACE, Jackknife, Shannon, and Simpson) and taxonomic profile were screened using EzBiocloud 16S-based MTP software (Yoon et al. 2017).

### 2.5. Statistical analysis

Data were statistically analyzed using GraphPad Prism 8 (GraphPad Software Inc., San Diego, CA, USA), including one-way ANOVA and Tukey’s multiple comparison test. Results were presented as mean ± standard error of the mean (SEM), with *p*** **<** **0.05 denoting statistical significance.

## 3. Results

### 3.1. Inactivation of L. reuteri PSC102

**Figure 1.**
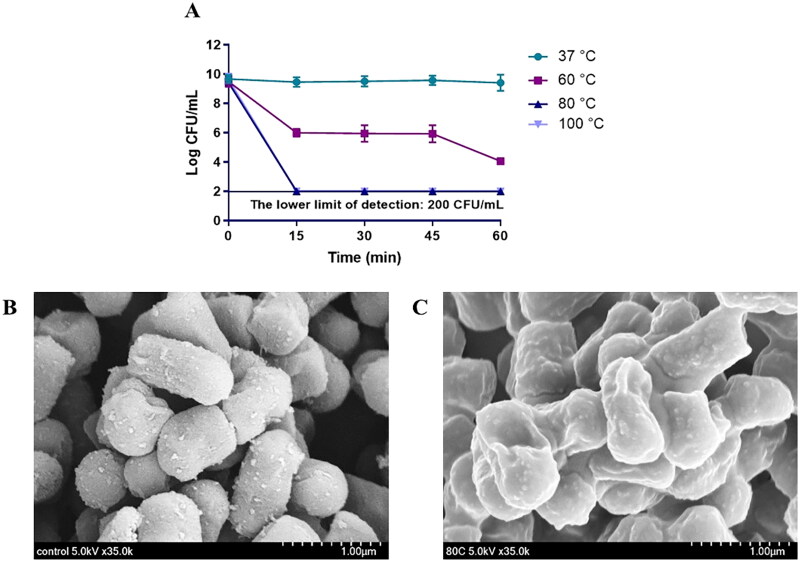
(A) *L. reuteri* PSC102 was killed at different temperatures (37 °C, 60 °C, 80 °C, and 100 °C) and time points (0, 15, 30, 45, and 60 min). Inactivation was measured by determining the colony forming units per milliliter at 15 min intervals after heat treatment. SEM image of (B) viable and (C) heat-inactivated *L. reuteri* PSC102.

To obtain hLR, we heated *L. reuteri* PSC102 at different temperatures and time points. The results showed that the loss of viability occurred when the bacteria were subjected to heat treatment at 80 °C for 15 min (Figure 1A). After heat inactivating *L. reuteri* PSC102, we determined its morphological characteristics *via* SEM. As shown in Figure 1C, heat treatment (80 °C for 15 min) killed the bacteria, leaving it with slightly unsmooth surfaces. We also obtained the inactivated *L. reuteri* PSC102 ghost (LRG) by treating it with the minimum bactericidal concentration (MBC) of NaOH (Table S1), which created pores in its structure (Figure S2).

### 3.2. RAW264.7 cell activation by hLR and LR treatments

**Figure 2.**
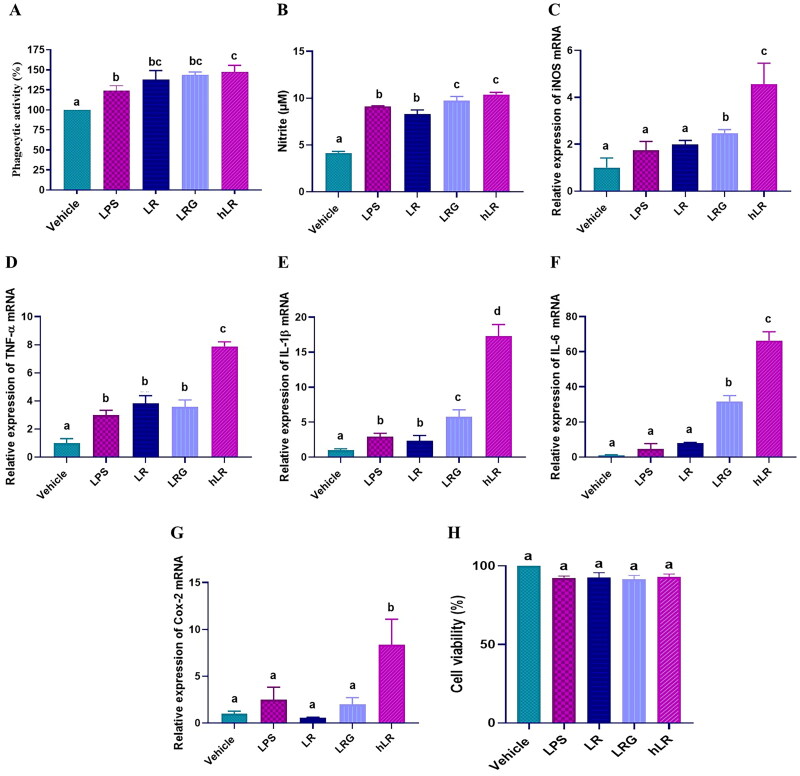
Effects of LR, LRG, and hLR on phagocytic activity, nitric oxide (NO) production, and cytokine expression. RAW264.7 macrophage cells were treated with LR, LRG, and hLR for 24 h. Phagocytic activity was determined by neutral red uptake assay (a). NO production was measured using griess reagent (B), and cytokines were measured *via* qRT-PCR: (C) iNOS, (D) TNF-α, (E) IL-1β, (F) IL-6, and (G) Cox-2. (H) Cell viability. PBS (vehicle), negative control; LPS (100 ng/mL), positive control; LR, live *L. reuteri* PSC102 (10^9^ CFU/mL); LRG, *L. reuteri* PSC102 ghost (10^9^ CFU/mL) and hLR, heat-killed *L. reuteri* PSC102 (10^9^ CFU/mL). Data are presented as mean ± SEM (*n* = 3). Different letters (a–d) above the bars indicate significant differences (*p* < 0.05) between groups.

We determined whether the hLR, LRG, and LR treatments could affect the activity of RAW264.7 cells. Upon treatment, the phagocytic activity and the expression of iNOS, IL-1β, TNF-α, IL-6, and Cox-2 significantly increased in RAW264.7 cells (*p*** **<** **0.05; Figure 2). The production of NO was also significantly induced (*p*** **<** **0.05; Figure 2B). Moreover, hLR, LRG, and LR treatment did not exert any cytotoxic effect on RAW264.7 murine macrophage cells (Figure 2H).

### 3.3. In vivo immunomodulatory effects in rats

For the animal experiments, hLR and LR were selected on the basis of *in vitro* results. The samples were orally administered to Sprague–Dawley rats for 3 weeks and assessed whether the immunostimulatory activities could be replicated *in vivo*.

#### 3.3.1. Body weight analysis

**Figure 3.**
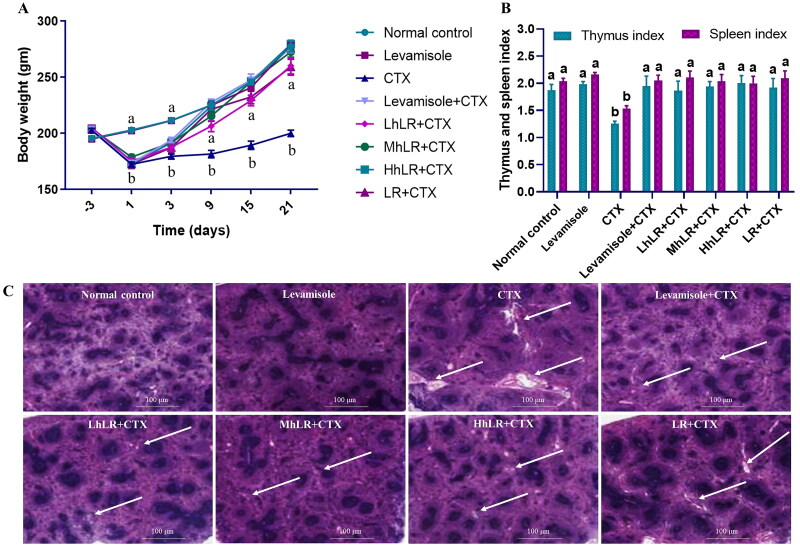
Effects of hLR and LR on body weight (a), thymus and spleen indices (B), and spleen tissues in CTX-treated rats (C). Spleen tissues were stained with H&E for histomorphology images (observed at 200×; scale bar = 100 μm). The white arrows indicate necrotic lesions. Data are expressed as mean ± SEM (*n* = 8). Significant differences (*p* < 0.05) between the groups are indicated with different letters (a–b) in superscript.

Throughout the experiment, the body weight of the rats in all eight groups was measured regularly (Figure 3A). No significant changes in the initial body weight of the rats were observed during the acclimatization period. After the rats were i.p. injected with CTX, the body weight of the six immunosuppressed rat groups was significantly lower than that of the control group (*p*** **<** **0.05). However, throughout the remaining study period, the body weights of the hLR (L, M, and H), LR, and positive control groups increased compared with those of the CTX group.

#### 3.3.2. Immune organ indices

The immune organ (thymus and spleen) indices were effectively reduced in the CTX-treated group compared with those in the control group (*p*** **<** **0.05; Figure 3B). However, the thymus and spleen indices were considerably higher in the hLR and LR treatment groups than in the CTX group (*p*** **<** **0.05).

#### 3.3.3. Histological observation of the spleen

Immune system destruction is accompanied by damage to the immune organs. In the present study, the ultrastructure of the spleen was examined by H&E staining to further examine its histomorphological characteristics. The spleen of the normal control group displayed tight and closely arranged spleen cells with no lesions (Figure 3C). In the CTX treatment group, larger necrotic areas were observed in the H&E-stained histopathological images of the spleen cells. However, in the hLR and LR treatment groups, the ultrastructure of the spleen cells showed no lesions, and this finding was comparable with that of the control group. Therefore, hLR and LR treatments could prevent CTX-induced spleen damage in rats.

#### 3.3.4. Effects of hLR and LR on immune cell indices in immunosuppressive rats

To evaluate the effect of hLR and LR on blood immune cells, we determined the number of immune cells in CTX-treated immunosuppressive rats. The WBC, lymphocyte, granulocyte, and MID counts in the CTX group were significantly lower than those in the control group (*p*** **<** **0.05). However, hLR and LR treatments significantly recovered the CTX-induced decrease in WBC, lymphocyte, granulocyte, and MID counts (*p*** **<** **0.05; Table 2).

**Table 2.** Effects of hLR and LR on blood cell counts in CTX-induced immunosuppressive rats.

Groups	WBC (10^9^/L)	Lymphocyte (10^9^/L)	Granulocyte (10^9^/L)	MID (10^9^/L)
Normal control	5.82 ± 0.33^a^	5.10 ± 0.15^a^	5.05 ± 0.27^a^	5.80 ± 0.17^a^
Levamisole	5.05 ± 0.14^a^	3.82 ± 0.28^ab^	4.02 ± 0.71^a^	5.45 ± 0.26^a^
CTX	1.82 ± 0.10^b^	1.67 ± 0.18^c^	2.60 ± 0.24^b^	2.35 ± 0.07^b^
Levamisole + CTX	4.10 ± 0.09^c^	2.30 ± 0.09^bc^	4.25 ± 0.21^a^	4.55 ± 0.17^a^
LhLR + CTX	4.37 ± 0.05^c^	2.87 ± 0.21^b^	4.50 ± 0.16^a^	5.00 ± 0.22^a^
MhLR + CTX	4.52 ± 0.16^c^	4.32 ± 0.45^a^	4.57 ± 0.29^a^	5.57 ± 0.39^a^
HhLR + CTX	5.22 ± 0.06^a^	4.77 ± 0.50^a^	4.87 ± 0.37^a^	5.80 ± 0.37^a^
LR + CTX	4.55 ± 0.12^c^	3.12 ± 0.31^b^	4.50 ± 0.23^a^	5.70 ± 0.15^a^

Data are expressed as mean ± SEM (*n* = 4). Significant differences (*p* < 0.05) between groups are indicated with different letters (a–c) as superscripts.

#### 3.3.5. Effects of hLR and LR on neutrophil migration and phagocytosis

**Figure 4.**
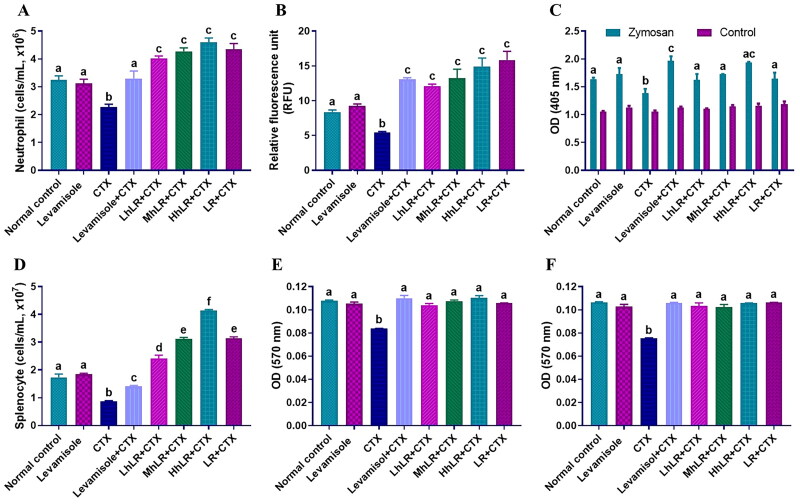
Effects of hLR and LR on neutrophil migration and phagocytosis, and splenocyte proliferation in CTX-induced immunosuppressive rats. A) Total neutrophils count in different groups. B) Neutrophil migration and C) phagocytosis. D) Total splenocyte count in different groups. *Ex vivo* splenocyte proliferation induced by Con A (E) and LPS (F). Data are expressed as mean ± SEM (*n* = 4). Different letters (a–f) above the bars indicate significant differences among the groups (*p* < 0.05).

To determine the primary functions of immunity, we assessed the neutrophil migration and phagocytic activities, because neutrophils are potent effector cells that are usually transferred to the site of infection to counteract foreign invaders (Rosales et al. 2017). As shown in Figure 4A, the hLR- and LR-treated rats showed a significantly higher neutrophil count than the CTX-treated rats did. Additionally, CTX treatment decreased the migration (Figure 4B) and phagocytosis (Figure 4C) of neutrophils (*p*** **<** **0.05). However, hLR (L, M, and H) and LR overturned the impaired migration activity of neutrophils (*p*** **<** **0.05; Figure 4B). We also examined the neutrophil phagocytosis activity by using zymosan as a substrate and found that the phagocytic activity of the neutrophils of the hLR- and LR-treated rats significantly increased (*p*** **<** **0.05; Figure 4C).

#### 3.3.6. Effects of hLR and LR on primary splenocyte proliferation

Splenocyte proliferation can be used to measure cellular immunity since immunological activation is characterized by immune cell proliferation (Heinzel et al. 2018). Splenocytes typically contain various cell types, including macrophages, T lymphocytes, and B lymphocytes. In the present study, the hLR- and LR-treated rats displayed significantly increased spleen cells compared with those in the CTX-treated rats (Figure 4D). Moreover, the proliferation of spleen lymphocytes to mitogen Con A (Figure 4E) and LPS (Figure 4F) stimuli was significantly suppressed in the CTX-treated rats compared with that in the normal control group. However, hLR and LR treatments significantly increased the splenocyte proliferation in the CTX-treated rats (*p*** **<** **0.05).

#### 3.3.7. Effects of hLR and LR on the expression of PBMC T cells

**Figure 5.**
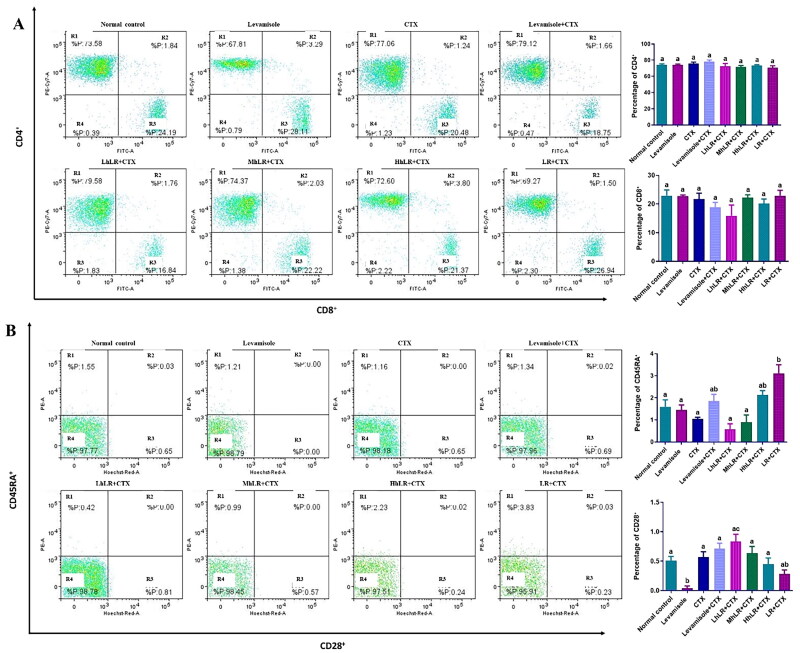
Effects of hLR and LR on PBMC T lymphocyte subsets in CTX-treated rats. A) Percentage of CD4^+^ (R1 region) and CD8^+^ (R3 region) T cell population. B) Percentage of CD45RA^+^ (R1 region) and CD28^+^ (R3 region) T cell population. Data are expressed as mean ± SEM (*n* = 4). Different letters (a–c) above the bars indicate significant differences (*p* < 0.05) between groups.

We used flow cytometry to analyze the effects of hLR and LR on the expression of T cells in PBMCs (Figure 5). hLR and LR treatments did not significantly affect the expression of CD4^+^ and CD8^+^ T lymphocytes compared with that of the CTX treatment. The CD4^+^ and CD8^+^ levels in all the groups were 70%–80% and 20%–30%, respectively (Figure 5A). In CD45RA^+^ and CD28^+^ differentiation, HhLR and LR treatments increased the CD45RA^+^ level but not the CD28^+^ level (Figure 5B).

#### 3.3.8. Effects of hLR and LR on the expression of thymic T cells

**Figure 6.**
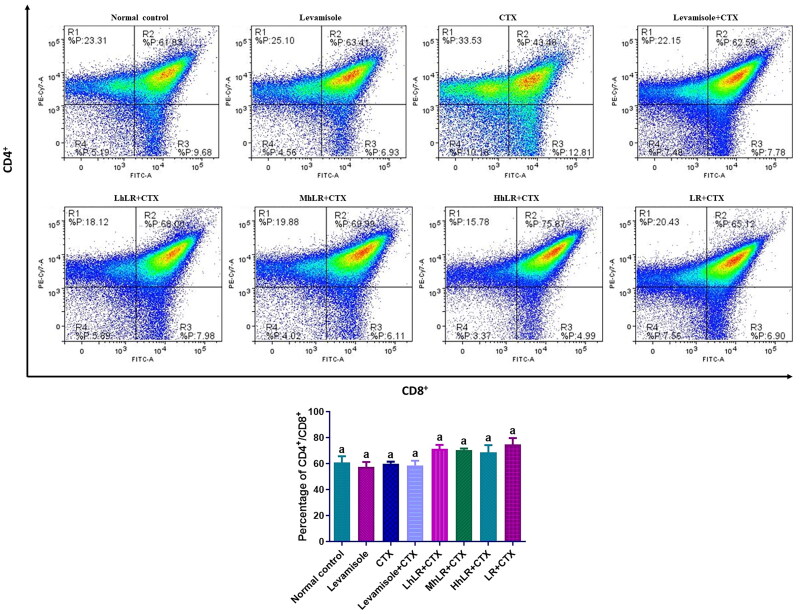
Effects of hLR and LR on thymic T lymphocyte subsets in CTX-treated rats. Percentage of CD4^+^/CD8^+^ (R2 region) T cell population. Data are expressed as mean ± SEM (*n* = 4). Different letters above the bars indicate significant differences (*p* < 0.05) between groups.

The effects of hLR and LR on the expression levels of thymic T cells are shown in Figure 6. The results revealed that CD4^+^ and CD8^+^ T cell expression was not significantly suppressed by CTX treatment. The high population of lymphocytes was double positive. However, hLR and LR treatments did not significantly change the expression levels of CD4^+^ or CD8^+^ T cells.

#### 3.3.9. Effects of hLR and LR on the expression of splenic T cells

**Figure 7.**
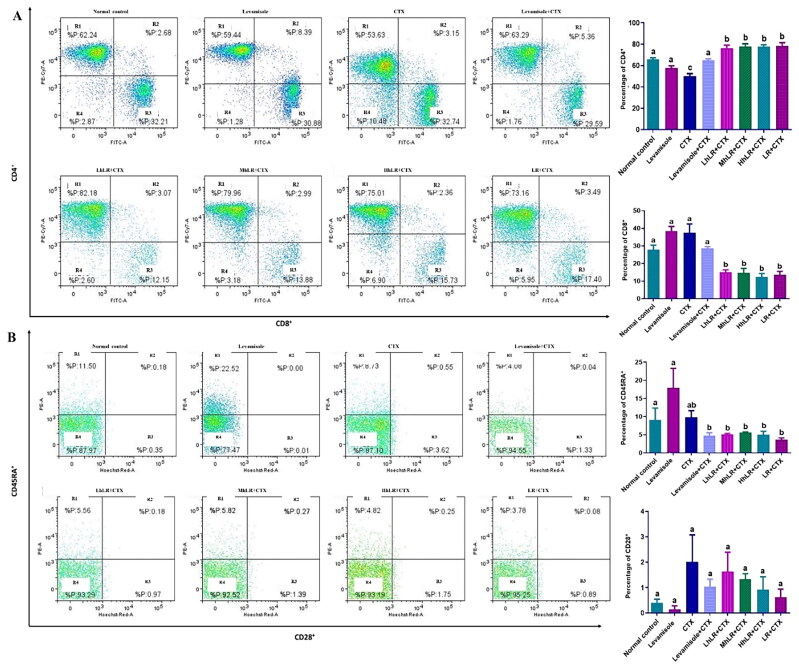
Effects of hLR and LR on splenic T lymphocyte subsets in CTX-treated rats. A) Percentage of CD4^+^ (R1 region) and CD8^+^ (R3 region) T cell population. B) Percentage of CD45RA^+^ (R1 region) and CD28^+^ (R3 region) T cell population. Data are expressed as mean ± SEM (*n* = 4). Different letters (a–b) above the bars indicate significant differences (*p* < 0.05) between groups.

The effects of hLR and LR on the expression of T cells in the spleen are presented in Figure 7. The percentage of CD4^+^ in the splenocytes of hLR- and LR-treated groups was significantly higher than that of the CTX-treated group (*p*** **<** **0.05; Figure 7A). hLR treatment might favor the differentiation of lymphocytes into CD4^+^ helper T cells (more than 75%) compared with that of their differentiation into CD8^+^ cytotoxic T cells (12%–15%; Figure 7A). In CD45RA^+^ and CD28^+^ differentiation, the CD45RA^+^ expression significantly decreased in the hLR and LR treatment groups, but the CD28^+^ expression was not statistically significant (Figure 7B).

#### 3.3.10. Effects of hLR and LR on cytokine expression

The concentrations of the cytokines IL-2, IL-4, IL-6, interferon (IFN)-γ, TNF-α, IL-10, and IL-12A in the serum of the rats were analyzed to assess the effects of hLR and LR on cytokine production. As shown in Table 3, the CTX treatment significantly reduced the levels of cytokines compared with those in the control group (*p*** **<** **0.05). However, our findings demonstrated that all cytokine levels in the hLR (L, M, and H) and LR treatment groups were significantly higher than those in the CTX group (*p*** **<** **0.05). Interestingly, hLR could more effectively reverse the CTX-induced attenuation of cytokine expression than LR.

**Table 3.** Effects of hLR and LR on cytokine levels in serum obtained from CTX-treated rats.

Groups	IL-2 (pg/mL)	IL-4 (pg/mL)	IL-6 (pg/mL)	IFN-γ (pg/mL)	TNF-α (pg/mL)	IL-10 (pg/mL)	IL-12A (pg/mL)
Normal control	20.68 ± 0.77^a^	121.28 ± 4.45^a^	100.44 ± 1.05^a^	145.13 ± 0.84^a^	169.17 ± 11.99^a^	36.90 ± 1.26^a^	165.65 ± 7.14^a^
Levamisole	25.13 ± 0.68^b^	127.58 ± 3.82^a^	113.84 ± 4.17^b^	158.06 ± 2.41^b^	177.65 ± 6.72^a^	40.65 ± 3.42^a^	212.50 ± 5.76^b^
CTX	17.42 ± 0.31^c^	84.86 ± 2.78^b^	72.75 ± 2.43^c^	126.43 ± 1.09^c^	111.89 ± 5.52^b^	26.11 ± 1.54^b^	121.97 ± 5.52^c^
Levamisole + CTX	25.39 ± 0.57^b^	154.21 ± 2.87^c^	119.87 ± 3.08^b^	159.15 ± 3.31^b^	188.30 ± 6.92^a^	47.81 ± 1.90^c^	267.36 ± 6.56^d^
LhLR + CTX	22.26 ± 0.55^a^	148.89 ± 2.34^c^	134.93 ± 5.96^d^	149.91 ± 2.48^a^	214.71 ± 3.53 ^cd^	34.52 ± 0.60^d^	227.89 ± 4.15^b^
MhLR + CTX	25.57 ± 0.43^b^	171.82 ± 4.05^d^	143.58 ± 3.32^d^	170.02 ± 3.23^d^	228.08 ± 5.47^c^	41.79 ± 1.10^a^	309.34 ± 1.80^e^
HhLR + CTX	29.71 ± 0.30^d^	175.08 ± 5.06^d^	147.75 ± 2.58^d^	184.15 ± 1.19^e^	234.93 ± 3.25^c^	51.68 ± 1.96^e^	330.65 ± 7.06^e^
LR + CTX	21.86 ± 0.53^a^	162.91 ± 2.60^c^	129.74 ± 1.75^bd^	161.54 ± 7.85^bd^	208.52 ± 5.48^d^	40.20 ± 2.67^a^	279.86 ± 6.53^d^

Data are expressed as mean ± SEM (*n* = 4). Significant differences (*p* < 0.05) between groups are indicated with different letters (a–e) as superscripts.

#### 3.3.11. Effects of hLR and LR on gut microbiota modulation

**Figure 8.**
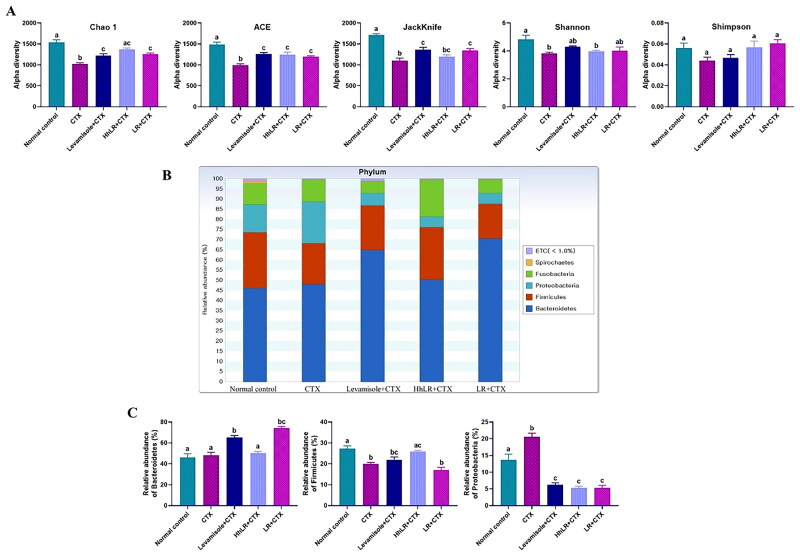
Effects of hLR and LR administration on gut microbiota. (A) Alpha-diversity assessed by Chao 1, ACE, Jackknife, Shannon, and Simpson indices. (B) Composition of gut microbiota at the phylum level. (C) The relative abundance of Bacteroidetes, firmicutes, and proteobacteria. Data are presented as mean ± SEM (*n* = 3). Different letters (a–c) above the bars indicate significant differences (*p* < 0.05) between groups.

**Figure 9.**
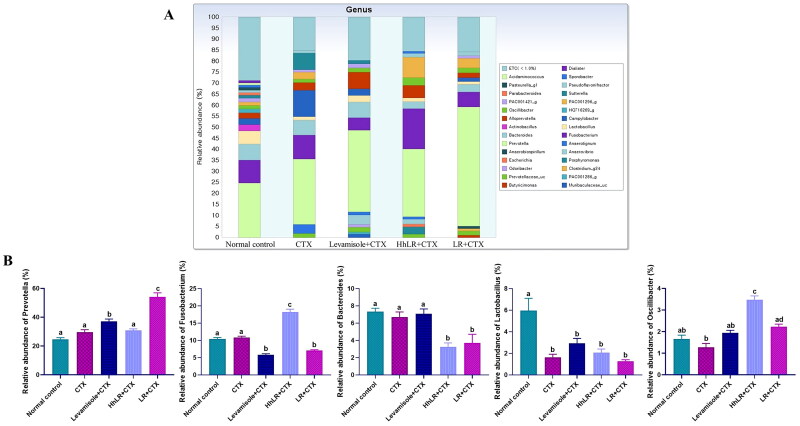
Effects of hLR and LR on the intestinal microbiota taxonomic composition. (A) Composition of gut microbiota at the genus level. (B) The relative abundance of *Prevotella*, *Fusobacterium*, *Bacteroides*, *Lactobacillus*, and *Oscillibacter*. Different letters (a–d) above the bars indicate significant differences (*p* < 0.05) between groups.

In our study, 449,248 high-quality bacterial gene sequences were obtained from five groups through high-throughput 16S rRNA gene sequencing analysis. The alpha-diversity of the gut microbiota was determined in terms of Chao 1, ACE, Jackknife, Shannon, and Simpson indices (Figure 8A). The community richness can be evaluated using Chao 1, ACE, and Jackknife, while the community diversity in the samples can be assessed using Shannon and Simpson indices. hLR and LR treatments significantly reversed the decrease in Chao 1, ACE, and Jackknife indices in the CTX-treated rats (*p*** **<** **0.05). The Shannon and Simpson indices increased in the treatment groups, but this increase was not significantly different from that in the CTX group. Taxonomic analysis revealed that Bacteroidetes and Firmicutes were the most abundant gut microbiota, and *Prevotella*, *Fusobacterium*, *Bacteroides*, and *Lactobacillus* were the most abundant genera in all the groups (Figure 8B, C, 9A, B). hLR treatment significantly increased the abundance of Bacteroidetes, while LR treatment significantly increased the level of Firmicutes compared with that in the CTX-treated groups. However, the levels of Proteobacteria in all treatment groups were decreased compared to CTX-treated groups (Figure 8B, C). The taxonomic profiles indicated that the compositions of *Prevotella*, *Fusobacterium*, *Lactobacillus*, and *Oscillibacter* were modulated by hLR and LR treatments (Figure 9A).

## 4. Discussion

Probiotics offer a wide range of positive health effects on humans and other animals. Although evidence supporting the use of live probiotics for clinical practices in many countries is widely available, recent studies have suggested that non-viable probiotics containing bioactive compounds may have many advantages over viable ones (Piqué et al. 2019). While live probiotics may encounter challenges in adhering to intestinal epithelial cells, which is primarily attributed to the presence of the mucous layer, which acts as a barrier preventing direct contact between bacteria and epithelial cells. However, it is worth noting that non-viable bacteria and their products have the ability to traverse the mucus layer in a more direct manner (Taverniti and Guglielmetti 2011). Moreover, the inactivated bacteria do not provide infection and have less possibility of transferring antibiotic resistance. The supernatant of cell-free culture and various soluble components present in probiotic culture media, including metabolites and other substances that are produced, cross the mucous barrier and engage with the mucosal immune cells within the intestinal monolayer (Bermudez-Brito et al. 2013). The metabolites produced by *Lactobacillus* exhibit antioxidant and anti-inflammatory characteristics, exerting their effects primarily on the cells lining of the intestinal mucosa and, subsequently, the immune system (De Marco et al. 2018). In relation to immunity, it is commonly observed that most bacteriocin-producing strains possess an immunity mechanism that entails the presence of this immunity protein (Pérez-Ramos et al. 2021). Probiotic *Lactobacillus* can produce a variety of short-chain fatty acids (SCFAs), such as lactic, formic, butyric, and propionic acids, which have been shown to have immunomodulatory properties (Parada Venegas et al. 2019). The bioactive peptides synthesized by *Lactobacillus rhamnosus* 1.0320 can stimulate lymphocyte proliferation, enhance phagocytosis and macrophage maturation, and promote splenic lymphocyte proliferation (Gao et al. 2021). The immunomodulatory effects of probiotic lactobacilli have garnered significant attention from researchers, mostly due to their ability to produce EPSs. The evidence indicates that the EPSs released by *L. acidophilus* DSMZ 20079 have the potential to enhance immune activities through the modulation of cytokines such as IL-2, IL-8, and TNF-α levels (El-Deeb et al. 2018). A prior investigation demonstrated that the lipoteichoic acids derived from *L. plantarum* have the capability to elicit the production of IL-12, consequently promoting the activation of innate immunity in the culture of splenic dendritic cells in mice (Hirose et al. 2010). It was revealed that the peptidoglycan obtained from *L. rhamnosu*s exhibited the ability to enhance the innate immune response in immunocompromised mice infected with *Streptococcus pneumoniae* (Barbieri et al. 2017). Surface-layer (S-layer) proteins are composed of proteins and glycoproteins that envelop the cellular surface in different strains of probiotic bacteria. The capacity of the S-layer protein found in *L. acidophilus* to bind to dendritic cells results in the development of an immunoregulatory phenotype known as Treg. This interaction also contributes to the maintenance of mucosal homeostasis, which has been associated with the ability of probiotic bacteria to induce an immunological response through their binding to dendritic cells (Klotz et al. 2020). However, consuming live probiotics may create problems for neonates (Navarro-Tapia et al. 2020) and vulnerable patients (Goldenberg et al. 2017) mainly because bacteria can move from the gastrointestinal tract to systemic circulation, causing infections and creating antibiotic resistance (Imperial and Ibana 2016). Conversely, consuming heat-inactivated probiotics is safer than taking live probiotics (Chen et al. 2017); the former also offers a lower risk of antibiotic resistance, exerts biological responses, and restores normal intestinal homeostasis (Piqué et al. 2019). Heat-killed probiotics can also provide other benefits, such as extending shelf-life and reducing reactions with other materials (Bernardeau and Cretenet 2019). Therefore, the proper selection and subsequent inactivation of probiotics can contribute to the development of new functional foods.

To produce hLR, we first treated the bacteria at different temperatures, and their viability was assessed by counting their CFU per milliliter in MRS media. After confirming the inactivation, we performed SEM analysis and found that the heat inactivation of *L. reuteri* PSC102 at 80 °C for 15 min left the bacteria with a slightly unsmooth surface. A previous study showed that applying optimum temperature to kill bacteria may produce a slightly uneven surface that can still produce immunomodulatory effects (Ou et al. 2011). Then, we used the heat-killed and live *L. reuteri* PSC102 to investigate the immunomodulatory effects on RAW264.7 cells and Sprague–Dawley rats.

The stimulation of RAW264.7 cells with hLR significantly increased the phagocytic capability and the expression levels of iNOS, IL-6, TNF-α, IL-1β, and Cox-2. The significance of macrophage phagocytic activity in host defense against pathogens is underscored, as it serves as a pivotal component of immunity. Macrophages possess the ability to engulf senescent cells, aberrant cells, and invasive pathogens, thereby augmenting the body’s capacity to combat infections *in vivo*. Hence, phagocytosis stands as a critical function executed by macrophages (Sueiro-Benavides et al. 2021). The upregulation of the cytokine expression is an indicator of immunostimulatory effects (Azad et al. 2018). Similarly, hLR increases NO production, one of the most versatile participants in the immune system, which can also cause macrophages to inhibit pathogen replication (Luo et al. 2023). Interestingly, hLR displayed higher stimulatory effects on the expression of cytokines and the production of NO than live LR and LRG did. In short, hLR stimulation enhanced RAW264.7 phagocytic activity, likely correlated with elevated nitric oxide (NO) levels and upregulated cytokine expression. In this study, we used heat-killed whole cell lysates to determine the bioactivity of hLR, which could potentially be used as a functional food/feed supplement. Therefore, considering the practical use of hLR in the food sector, we selected whole cell lysates. *L. reuteri* PSC102 has various structural components, such as teichoic acid, lipoteichoic acids, and peptidoglycans, which may contribute to immunomodulatory effects. Previous studies showed that the cytoplasmic portion and cell wall components of probiotics play immunomodulatory roles (Taverniti and Guglielmetti 2011).

CTX is a well-known cytotoxic drug used in cancer treatment. However, CTX as an anticancer agent produces many side effects, including immune organ atrophy and weight loss, bone marrow suppression, and an imbalance in immune blood cells, ultimately inhibiting immune functions (Wang et al. 2011). Moreover, administration of CTX can interfere with the proliferation and differentiation of T and B cells as a result of the disruption of the T helper (Th)1/Th2 balance (Yu et al. 2015). Hence, to evaluate the immunostimulatory effects of *L. reuteri* PSC102, rats treated with CTX were used as an immunosuppressed model.

We first assessed body weight, immune organ indices, spleen morphology, and immune blood cell counts. The obtained data revealed that hLR and LR treatment diminished the CTX-induced decrease in body weight, thymus and spleen indices, splenocyte proliferation, and neutrophil phagocytosis. The spleen is among the major organ maintaining immune homeostasis (Cui et al. 2016). The H&E results of the spleen indicated that hLR and LR could inhibit the CTX-induced damage on the spleen, revealing that hLR and LR could reverse the effect of immunosuppression in rats. Moreover, the total number of WBCs, lymphocytes, granulocytes, and MID increased compared with that in the CTX-treated rats upon administration of hLR and LR, thereby providing corroborative evidence for their immunostimulatory effects.

The ability of neutrophils to migrate to the site of foreign invaders and subsequently phagocytose them is essential for maintaining host immunity. The neutrophil migration and phagocytosis activity in hLR- and LR-treated groups improved compared with those in the CTX group, indicating that hLR and LR played a vital role in initiating and modulating nonspecific immunity; thus, the immunity of immunosuppressed rats has been improved. Lymphocyte proliferation in response to mitogen is commonly used to determine the efficiency of immunomodulatory agents; therefore, the proliferation of lymphocyte assay has been used to evaluate the effect of probiotics on immune function (Maroof et al. 2012). To determine the effect of hLR and LR on cellular immunity, we isolated splenocytes from rats and examined their proliferative activity. The results demonstrated that hLR and LR treatments significantly augmented splenocyte proliferation, suggesting that they could improve humoral and cell-mediated immunity.

Hematopoietic stem cells (HSCs) usually stay in the bone marrow, differentiate into different cell types, and mobilize to the periphery. T cell progenitors move from the bone marrow into the systemic circulation during development and then enter the thymus and spleen (Kisielow 2019). Our results showed that the population of thymic T lymphocytes, also known as thymocytes, appeared to be double positive (CD4^+^ and CD8^+^), as positive and negative selection have not taken place as part of the maturation process. From the representative dot plots, double-positive thymocytes constituted the majority of the total cell population, regardless of treatment. In the spleen, the total population of splenic T lymphocytes, commonly known as splenocytes, comprises single positive cells expressing either CD4 or CD8. Analysis of the hLR and LR-treated groups reveals an augmented CD4^+^ splenocyte population and a reduced CD8^+^ splenocyte population, suggesting potential impacts on CD4/CD8 lineage differentiation due to the treatment. The critical factor influencing lineage selection during T-cell development is the duration of the T-cell receptor (TCR) signaling. Prolonged signals, exemplified by extended exposure to hLR and LR treatment, favor CD4 lineage commitment, while transient signals favor CD8 lineage commitment (Steier et al. 2023).

In the immune system, Th1 and Th2 maintain humoral and cellular immunity. Cytokines also have a crucial role in improving immunological responses by regulating T cell proliferation and differentiation. IL-2 is an essential cytokine that helps in the survival and proliferation of T cells (Meng et al. 2018). Hence, hLR and LR treatment increased the release of IL-2, which might encourage the proliferation of T cells and the expression of IFN-γ, ultimately improving immune responses. The release of TNF-α and IL-12A is upregulated upon the treatment of *Lactobacillus*, which may help boost immunity; this finding corroborates our results (Jorjão et al. 2015). IL-6, secreted by Th2 cells, can regulate humoral immunity and modulate T cell proliferation and differentiation (Jang et al. 2013). IFN-γ helps differentiate Th cells into Th1, such as CD4^+^ T cells and CD8^+^ T cells, contributing to cellular immune responses (Chang et al. 2008; Li et al. 2013). Similarly, IL-4 stimulates the differentiation of Th2 (Keegan et al. 2021). IL-10 promotes the differentiation of Tregs (Zhang and Kuchroo 2019). The Treg cell CD28^+^ is expressed on the T cell surface and required for T cell survival. CD28^+^ produces stimulatory signals, stimulating the production of T cells to recognize invading agents (Boomer and Green 2010); the immunoregulatory functions of CD45RA^+^ cells include the direct suppression of immune responses or the stimulation of a suppressive activity by CD8^+^ cells (Rocamora-Reverte et al. 2020). Therefore, we examined the expression of the cytokines in this study and demonstrated that hLR and LR could reverse the CTX-induced decrease in cytokine concentrations.

The gut microbiota can regulate intestinal functions and immune homeostasis, thereby keeping the balanced health of the host. CTX can change the composition of the gut microbiota, while probiotic administration can modulate the microbiota composition changed by CTX and thus regulate host immunity (Meng et al. 2019). Probiotics have the ability to generate metabolic products that exert modulatory effects on the proliferation of other bacteria in the gut. It was shown that EPS, an extracellular macromolecule released by probiotics, can regulate the abundance of the major phyla (Kaur and Dey 2022). Furthermore, EPSs derived from *L. rhamnosus* are being studied due to their modulating effects on the human gut microbiota (Zhu et al. 2021). The S-layer proteins of lactobacilli have been demonstrated to enable probiotics to bind to intestinal epithelium cells, thereby modulating the gut microbiome (Guli et al. 2021). The other probiotic effector molecules, such as peptidoglycan, teichoic acid, and bacteriocins, can modulate the gut microbiota and improve the homeostasis of the intestine (Gao et al. 2022). In the present study, the possible effects of hLR and LR on the gut microbiota in CTX-treated rats were investigated. The results demonstrated that hLR and LR treatments significantly increased the alpha-diversity index of the CTX-treated rats. Poor immune functions are typically linked to limited species and diversity (Rehman et al. 2010). Our results indicated that hLR and LR were advantageous for host health. To fully understand the similarities and differences between various groups, we compared the dominating gut microbiota at several taxonomic levels (phylum, family, and genus). The relative abundances of Bacteroidetes and Firmicutes were respectively increased by LR and hLR treatments in the CTX-treated rats. Bacteroidetes and Firmicutes may be involved in various strategies to rely on carbohydrate-active enzymes, thereby contributing to host immunity (Kato et al. 2014). Kim et al. (2020) has reported that treatment with *L. sakei* K040706 in CTX-induced immunosuppressed mice recovered Firmicutes and Bacteroidetes, positively impacting the immune response and gut health. The abundance of Proteobacteria significantly decreased in the sample treatment compared with those in the normal control and CTX-treated groups. This decrease in Proteobacteria may be beneficial for the gut health of the host because it is the major Gram-negative bacterial group comprising a wide range of pathogenic microorganisms, such as *Helicobacter pylori, E. coli*, and *Salmonella* (Zheng et al. 2017). *Prevotella* is one of the important members of gut microbiota, which was increased in the treatment groups. *Prevotella* is associated with cytokine release, which can modulate intestinal functional barriers (Han et al. 2021). Moreover, increasing other bacteria, such as *Lactobacillus* and *Oscillibacter*, may provide potential immunoregulatory roles. Gram-negative *Oscillibacter* mainly contains LPS and may trigger the release of cytokines through NF-κB activation (Ding et al. 2019). *Lactobacillus*, another important lactic acid bacteria, may play a crucial role in the immune system to provide healthy immune homeostasis in the host’s digestive tract (Han et al. 2021). *Lactobacillus* can also enhance gut mucosal immunity by stimulating TLR2 receptors in dendritic cells and macrophages (Tsai et al. 2010). Therefore, hLR and LR could modulate the gut microbiota composition, suggesting that the gut microbiota linked to immune responses may be involved in immunoregulation.

## 5. Conclusion

In this study, hLR and LR showed immunomodulatory effects by stimulating RAW264.7 cells. Furthermore, the oral administration of hLR and LR demonstrated the immunomodulatory effects in CTX-treated rats by promoting the development of immune organs and the hematopoietic system, increasing neutrophil migration and phagocytosis, changing lymphocyte proliferation and subpopulation expression, and upregulating cytokine levels. The intervention with hLR and LR modulated the gut microbiota composition, thereby increasing the relative abundance of Bacteroidetes and Firmicutes, which are positively linked to the immune response. Therefore, these results suggested that LR or hLR could be a potential immunostimulatory agent to improve immune functions in humans or other animals. However, further research is still required to elucidate the pharmacology active components in hLR and LR and signaling pathways related to immunomodulation.

## Supplementary Material

Supplemental Material

## Data Availability

The data obtained in the present study are available within the article and supplementary materials.
