# Supplementary material for: Modulating effects of heat-killed and live Limosilactobacillus reuteri PSC102 on the immune response and gut microbiota of cyclophosphamide-treated rats
Source: Vet Q. 2024 Apr 29;44(1):1–18. doi: 10.1080/01652176.2024.2344765 (PMC11060015; doi:10.1080/01652176.2024.2344765)
Supplement: Supplemental Material [file TVEQ_A_2344765_SM4595.docx]

Supplementary Figure 1. In vivo experimental schem


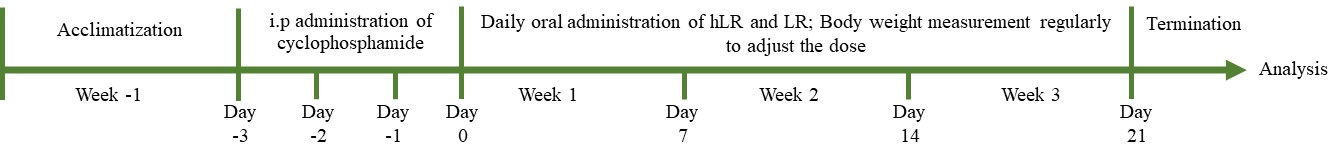


Supplementary Figure 2. (A) Inactivation of L. reuteri PSC102 at different concentration (1/3 MIC, MIC, 1/3 MBC, MBC) of NaOH with different time points (15, 30, 45, 60, and 75 min). Morphology of control L. reuteri PSC102 (B) and L. reuteri PSC102 ghost (LRG; C).


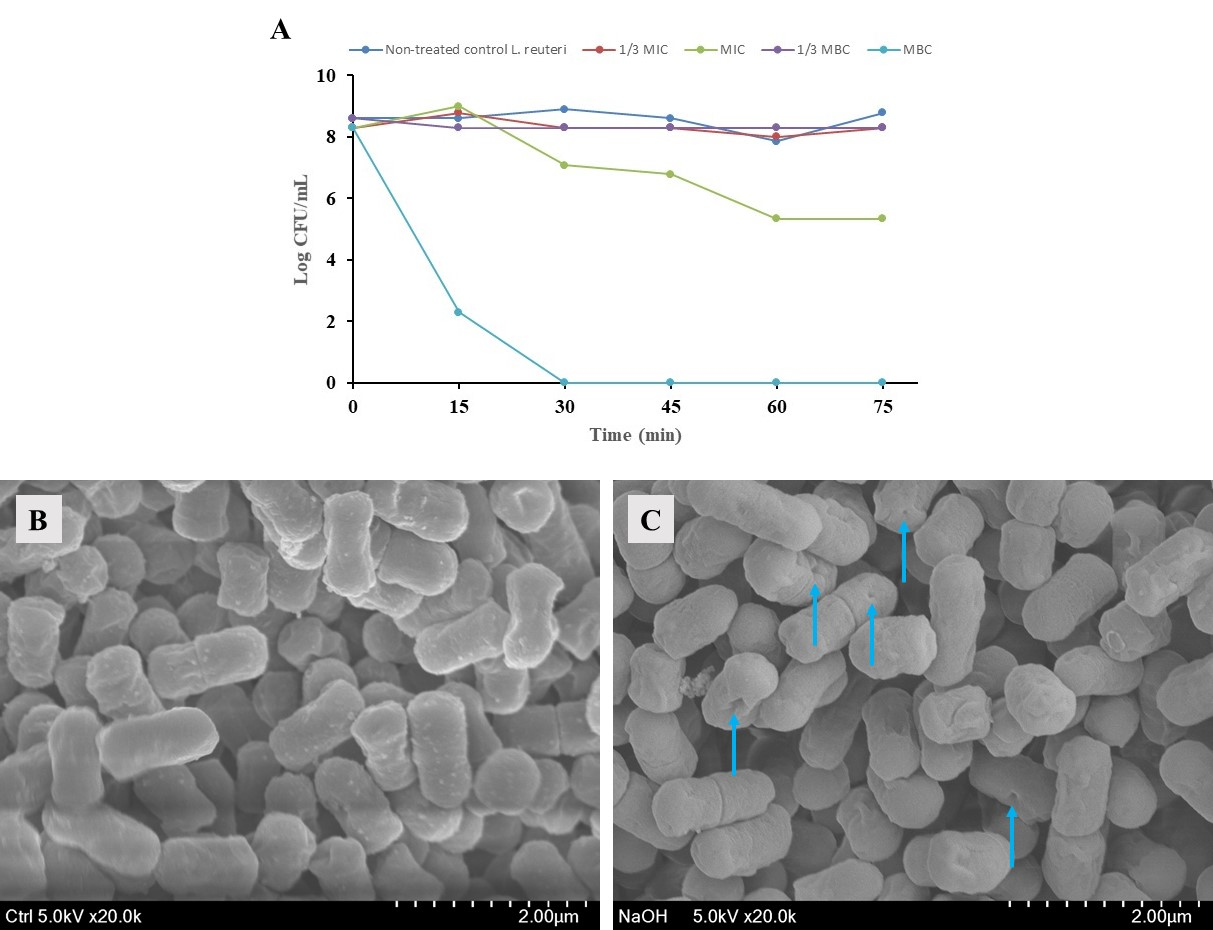


Supplementary Table 1. Minimum inhibitory concentration (MIC) and minimum bactericidal concentration (MBC) of different chemicals against *L. reuteri* PSC102

| SL No. | Chemicals | MIC (mg/mL) | MBC |
| --- | --- | --- | --- |
| 1 | NaOH | 1.875 | 2 × MIC |
| 2 | KOH | 3.75 | 2 × MIC |
| 3 | Sodium carbonate | 3.75 | 8 × MIC |
| 4 | Boric acid | Not detected | – |
| 5 | Citric acid | 3.75 | 8 × MIC |
| 6 | Methanol | Not detected | – |
| 7 | Hydrochloric acid | 7.5 | 2 × MIC |
| 8 | Sulfuric acid | 1.875 | 2 × MIC |
| 9 | Nitric acid | 3.75 | 2 × MIC |
| 10 | Acetic acid | 7.5 | 2 × MIC |
| 11 | Ethanol | Not detected | – |
| 12 | n-Butanol | Not detected | – |
